# Exploring linguistic and cultural adaptations to a functional behavior assessment interview with a teacher consultation case in Jordan

**DOI:** 10.3389/fpsyg.2026.1797698

**Published:** 2026-04-15

**Authors:** Billie Jo Rodriguez, Nibal AlHamouri, Felicia Castro Villarreal

**Affiliations:** 1 Special Education & Clinical Sciences, University of Oregon, Eugene, OR, United States; 2 Department of Educational Psychology, The University of Texas at San Antonio, San Antonio, TX, United States

**Keywords:** Arabic translation, cultural adaptation process, functional assessment interview, international behavioral consultation, school consultation in Jordan, teacher consultation, functional behavior assessment, culturally responsive interviewing

## Abstract

School consultation involves a specialist working with an educator to improve a student's or a group of students' learning and adjustment. However, the targets, processes, methods, and outcomes can vary globally. Functional Behavior Assessment (FBA) is a critical skill in school consultation that can support student behavioral outcomes. The Functional Assessment Checklist for Teachers and Staff (FACTS) is a commonly used semi-structured interview in teacher consultation but has not been studied cross-culturally or in global contexts. Though demand for behavior consultation is growing and school consultants can benefit from using the FACTS interview as part of the consultation problem-solving process in the Middle Eastern country of Jordan, cultural and linguistic considerations are needed to ensure supportive outcomes. This consultation case study describes a Critical Reflective Practice approach to the translation and adaptation of the FBA interview tool and process. In this case study, researchers integrate cultural adaptation models and multicultural and cultural humility frameworks to translate and conduct the FACTS interview in Arabic and to develop a hypothesis statement regarding student behavior in a Jordanian classroom. Consultation case study findings highlight the practice of researcher-practitioner reflexivity and considerations for cultural and linguistic adaptations to inform future translation and adaptation work in teacher consultation to support student outcomes in global school contexts.

## Introduction

Specialists can leverage school consultation as a powerful mechanism to improve the learning and adjustment outcomes of students as well as support educator skill development (Erchul and Martens, 2010). Increasing cultural and linguistic diversity has created a need for school consultation tools that can be used across language and cultural contexts to support academic and behavioral outcomes in educational settings. There is growing demand for assessment linked to intervention support in the United States (Behavior Analyst Certification Board, 2025) and across the globe (e.g., Al-Makahleh et al., 2024; Kingsdorf and Pancocha, 2023). Because most behavior assessment tools were developed in English, researchers and practitioners must translate them into other languages for global use. Traditional translation processes involve forward-back (FB) translation methods where one translates the tool into a new language and a second translator translates the tool back to the original language and then compares it to the original source document.

As demand for materials across global contexts increases, research has shown limitations in the FB translation alone (e.g., Canal-Bedia et al., 2011; Cuesta-Gomez et al., 2016; DuBay et al., 2021; DuBay and Watson, 2019; Magán-Maganto et al., 2018; McClure et al., 2018). Translating consultation tools is a necessary first step but is insufficient to recognize and meaningfully consider the broad and multidimensional nature of culture that extends well beyond language. Language alone cannot capture the nuanced cultural and historical contexts, values, and lived experiences that shape how people from different backgrounds engage with education. Although culture can influence one's preference and selection of assessment and intervention tools (Beaulieu and Jimenez-Gomez, 2022), issues regarding race, ethnicity, and culture are often overlooked (Fisher-Borne et al., 2015). In a review of research published in the Journal of Applied Behavior Analysis, less than 10% of studies reported participant race or ethnicity, and, when reported, Caucasian and African American made up 95% of participants (Jones et al., 2020). Representation and acknowledgment of different cultures are needed to inform consultation practices that can enhance students' wellbeing. Effective cross-cultural practices require a steep understanding of the target community's norms, beliefs, and educational practices that may not be fully reflected in linguistic translation. Cultural adaptation models and culturally responsive frameworks acknowledge cultural multidimensionality and overlap with social justice by considering systemic and structural forces that limit equitable access to resources and outcomes.

### Consultation & translation frameworks

The multicultural school consultation framework (Ingraham, 2000) sets the standard for multicultural consultation by centering culture, individuality, and diverse perspectives. The framework includes five components: (1) consultant knowledge and development (e.g., understanding one's own culture and impact, cultural context, individual differences within cultures, valuing other cultures, and appropriate considerations); (2) consultee learning and development (e.g., knowledge, skill, objectivity, and confidence); (3) cultural variations in the consultation constellation (e.g., consultant-consultee similarity, consultee-client similarity, and three-way diversity); (4) contextual and power influences (e.g., cultural similarity, society influences, and power balance disruptions); and (5) hypothesized methods for supporting consultee and client success (e.g., framing the problem and consultation process, multicultural consultation strategies, and continuing one's professional development; Ingraham, 2000). Recently, this framework has been enhanced by ideas of cultural responsiveness and multiculturalism that extend beyond race, ethnicity, and language (Goforth, 2020; Jones, 2009). Multiculturalism includes recognizing, valuing, and understanding cultural diversity while promoting equal opportunities (La Salle et al., 2020). Cultural responsiveness extends multiculturalism through the application and transformation of practices that align with individuals' values, beliefs, and cultural backgrounds (La Salle et al., 2020). Culturally Responsive (CR) consultation defines culture ecologically as broad, multidimensional, and intersecting, emphasizing alterable variables (e.g., behavioral antecedents and consequences) as well as identity, context, and culture when generating hypotheses and collaborating for solutions (Parker et al., 2020). Additionally, Critical Reflective Practice (CRP, Brookfield, 1995) incorporates systematic reflection on assumptions and power relations in practice. CRP involves a thoughtful review of dilemmas or moments of dissonance encountered during the process and encourages an analytical lens for reflection.

When language translation and test adaptation are necessary to support assessment within consultation, the International Test Commission (International Test Commission, 2017) provides guidelines that can be applied to consultation tools and methods such as interviews. These guidelines emphasize specific adaptations that align with the linguistic and cultural values of a target population. The ITC takes great care to detail their guidelines as more than traditional test translation guidelines, instead describing test adaptation guidelines as a broader methodology that refers to moving an assessment from one language and culture to another. According to the ITC, test adaptation includes determining whether a test or tool in another language could measure the same constructs, carefully selecting translators, considering accommodations, modifying test format, conducting the translations, and completing sophisticated statistical analyses to examine reliability and validity (International Test Commission, 2017). Linguistic translation is only part of that adaptation process.

The cultural humility framework extends the idea of cultural competence to incorporate a lifelong process of critical and systematic self-reflection that acknowledges existence in a multicultural world with power imbalances by centering optimal care through authentic relationships and lifelong learning (Fisher, 2020; Foronda et al., 2016), translating this to a “way of being.” Key attributes of the framework include being open, self-aware, egoless, choosing supportive interactions, and willingly engaging with diverse individuals to achieve mutual empowerment (Foronda et al., 2016). Cultural humility starts with assessing and acknowledging one's own beliefs, biases, and values. Next, one approaches others with openness, acceptance, and humility. Engaging in cultural humility (i.e., seeking others' points of view and valuing diverse perspectives and backgrounds within the context of a reciprocal and collaborative partnership) fosters trust and sets the stage for a more positive working alliance, effective problem-solving and outcomes, and reductions in consultant/consultee microaggressions (Fisher, 2020). The cultural humility framework posits that strong working relationships are built on the premise that cultural differences do not exist within any one person and instead exist in the interaction between people. This framework, and its emphasis on relational interaction, is especially relevant and foundational (Goforth et al., 2025) when interviewing educators as part of a functional behavior assessment (FBA) in school consultation.

### Functional behavior assessment in school consultation

FBA is an evidence-based approach to intervention development and supportive outcomes that involves operationally defining target behavior(s), analyzing environmental events that predict and maintain behaviors, and determining a hypothesis about why behaviors persist that can be used to inform effective and precise intervention development (O'Neill et al., 1997). FBA is an important skill in school consultation due to the effectiveness of function-based intervention development on outcomes (Ala'i-Rosales et al., 2019; Anderson et al., 2015; McIntosh and Av-Gay, 2007). One critical and foundational aspect of consultation, especially in the functional behavior assessment process, is interviewing to obtain stakeholder input regarding behaviors and environmental determinants. Interviews also provide stakeholder perspectives for feasible and supportive intervention practices. The Functional Assessment Checklist for Teachers and Staff (FACTS; March et al., 1999; McIntosh et al., 2008a,b) is an interview tool designed for use in school contexts, but it has not been studied cross-culturally or globally. Therefore, the implications of using the FACTS in an international, culturally diverse context are unknown. (Jessel et al. 2025) provide guidance for conducting culturally responsive and trauma-informed functional assessment interviews that is consistent with ITC guidelines and a cultural responsiveness framework. Their guidance includes (a) reflecting on one's own cultural background, (b) understanding a learner's lived experience, (c) engaging in cultural humility, (d) asking open-ended questions, (e) assessing social validity, (f) fostering collaboration, and (g) planning for generalization.

### Jordanian school context for behavioral consultation

One context with an emerging interest and need for school-based behavior consultation is the country of Jordan. In the United States (US), school psychologists are licensed to work in schools and commonly support students' academic, social, and mental health. Internationally, school-based mental health providers vary in terms of title, role, and function. In Jordan, for example, psychological counselors have various responsibilities and offer a range of services to address students' social, emotional, and behavioral needs (Al-Makahleh et al., 2024). They conduct neuropsychological, cognitive, and academic assessments to identify students' learning challenges and provide intervention support to help students manage emotional and social difficulties, enhancing overall wellbeing and academic performance (Abu Khudair et al., 2024). Recently, increasingly diverse behavior, academic, and social emotional needs of students in Jordanian schools has resulted in expanding the psychological counselor role to better support educators whose students exhibit learning and behavioral challenges (Al-Makahleh et al., 2024).

There is scarce research related to the application of school behavioral consultation practices to Jordanian contexts. The literature indicates limited availability of certified Applied Behavior Analysis (ABA) professionals, inadequate teacher training for implementation of applied behavior analytic evidence-based practices, and a reliance on punitive rather than positive reinforcement behavior support strategies in schools (Almutlaq, 2021). However, Middle Eastern countries, including Jordan and Saudi Arabia, have been intensifying efforts to enhance the quality of education for students with diverse learning, behavior, and mental health needs. In Saudi Arabia, for example, ABA practices are primarily confined to private clinics such as the Center for Autism Research (CFAR) (n.d.) in Riyadh and have not been formally integrated into the national special education system (Almutlaq, 2021). The Jordanian educational context is one that identifies the multi-faceted nature of student learning and behavior challenges, supporting a holistic approach (Al-Makahleh et al., 2024). Jordan officially introduced ABA in October 2005 when a delegation from the Association for Behavior Analysis International (ABAI) visited the country to promote behavior analysis. Since then, multiple programs incorporating the principles of applied behavior analysis have been adopted, primarily for children with autism in special settings (Khaleel, 2019). Therefore, some ABA influence is present in Jordan, and some teachers are applying behavior analysis to understand the function of student behavior (Almutlaq, 2021). However, this often occurs with limited resources, and teachers in Jordan and Saudi Arabia report difficulty linking data collection to practical intervention, highlighting the need for consultative support in functional behavior assessment and intervention (Almutlaq, 2021).

Currently, the only functional behavior assessment tool readily available in Arabic is the Functional Analysis Screening Tool (FAST, Iwata et al., 2013), which is a questionnaire used for screening caregivers and is not specific to school settings or for conducting FBA interviews. Although the FAST has been translated into Arabic, the translation process or outcomes have not been systematically analyzed. Recent research utilized an open-ended functional assessment interview (Alnemary et al., n.d.) where Arabic-speaking BCBAs interviewed caregivers in Saudi Arabia about their child's challenging behaviors and the related antecedents and reinforcers, conducted direct observations, and used this to inform hypothesis and interventions that were considered culturally appropriate and effective (Almarzooq et al., 2025). Despite these promising findings, the language and cultural translation process is not detailed or specifically analyzed. In addition, several non-school-based behavior rating scales have been translated into Arabic and systematically analyzed (i.e., the Hamilton depression rating scale, the hoarding rate scale, and the multidimensional cognitive attentional syndrome scale), typically following the FB translation approach. Across these studies, the researchers utilized independent translation by two individuals fluent in both languages, comparison and consensus on the translation, a review for linguistic clarity, accuracy, and cultural/religious appropriateness, and then back translation (Helmey et al., 2023; Hussain et al., 2023; Obeid et al., 2023). One step to meet the need for school-based Arabic FBA resources is to adapt school-based assessment and consultation tools for use in Jordan, integrating language translation best practices and culturally responsive frameworks.

This case study presents an exploratory descriptive teacher consultation case study conducted in Jordan in which the consultant functioned as a researcher-practitioner guided by practice-based knowledge and reflective inquiry to inform culturally responsive action. Consistent with guidance by Jessel et al. (2025), the consultation relied on Ingraham's Multicultural Consultation Model (Ingraham, 2000), a Cultural Humility Framework (Foronda et al., 2016), a cultural adaptation model (Bernal et al., 2009), and the International Test Commission's (2017) best practice guidelines to translate and adapt the FACTS interview from English to Arabic for use in Jordan. The researchers engaged in a researcher-practitioner case study to explore cross-cultural dilemmas and to reflect on how personal identity, language, and context may shape translation of the FACTS interview. The case study focuses on how the consultant's reflection of her personal identity, linguistic positioning, and sociocultural context influenced consultation. The consultant received training and supervision in school-based behavioral assessment and obtained systematic content and process input from two Jordanian teachers who were native Arabic speakers to develop an initial draft of a culturally and linguistically adapted FACTS interview in Arabic. As part of this process, the adapted FACTS interview tool was conducted with one of the Jordanian teacher participants working in a Jordanian private school. Consultee language translation and process feedback were then used to further refine and adapt the terms, interview questions, and process. This supported the development of an ecologically valid behavioral hypothesis statement to inform a contextually appropriate function-based intervention. Critical Reflective Practice (Brookfield, 1995) was central to this case study for both process and outcome data for comprehensive cross-cultural and culturally responsive consultation practice. The purpose of this descriptive teacher consultation case study is to share details illustrating an iterative process of using critical reflection and cultural humility to adapt the FACTS interview tool in a non-Western context. This may inform future translation and adaptation processes to advance knowledge regarding theory-to-practice considerations in cross-cultural assessment and consultation.

## Method

### Participants and settings

Three researchers and two teachers participated in the case study. The researchers were the graduate student consultant (i.e., consultant), a faculty member who provided clinical supervision of the case, and a third faculty member who engaged in critical consultation and review of consultation case materials. There were also two teacher participants who resided in Jordan. One served as an expert language and culture participant only, and the other served as the teacher consultee who provided language, content, and process feedback. The consultant was a Jordanian woman Native-Arabic speaker as well as a first-year special education graduate student living and attending a university in the northwestern US. She speaks and writes in Arabic and English and had 6 years of experience working as a math special education teacher in Jordan prior to beginning graduate training. She began in-person coursework in behavioral assessment and school consultation during the 6 months prior to engaging in the consultation case study and had no formal training in behavior theory or consultation before beginning graduate coursework in behavioral assessment and school consultation in the US. The clinical supervisor researcher for the consultant in the case is a full-time associate teaching professor in school psychology and special education at a university in the Pacific Northwest US, who served as the course instructor for the consultant and led the cultural and language translation work. She identifies as a white European American woman from a rural southern state with extensive school consultation experience. She is trained as a doctoral-level board-certified behavior analyst and is a nationally certified school psychologist. The third author is a full-time scholar at a large university in the Southwest US. She identifies as Mexican American and speaks and reads Spanish and English. She has extensive experience in the delivery of culturally responsive and socially just teacher consultation work and led the iterative and systematic reflective practice.

The consultant underwent in-person FBA and consultation training as part of her graduate program requirements prior to beginning the current consultation case work. She took graduate coursework on behavior theory, behavior assessment, evidence-based intervention strategies, and data collection methods. She also had coursework and practicum experience in school-based behavioral consultation, including role play and live feedback, conducting FACTS interviews, as well as demonstrating competency in supervised FBA casework in a school-based practicum experience. The consultation coursework also provided the consultant instruction in the cultural humility framework as a foundation for meaningful and authentic consultation work and training for applying the attributes of openness, self-awareness, egoless, supportive interactions, and self-reflection and critique (Foronda et al., 2016) as well as Critical Reflective Practice (CRP, Brookfield, 1995) to review moments of dissonance encountered and engage in reflective analysis.

The two teacher participants were recruited through a convenience sampling method and provided verbal consent to participate in the iterative translation and adaptation process as part of the consultation project. The consultant had prior professional experience working with them at a school in Western Amman, Jordan. Both teacher participants worked at a school providing bilingual education in Arabic and English using the International Baccalaureate (IB) program where the consultant had previously worked. The IB program is an inquiry-based educational framework that focuses on the holistic development of the student's social, emotional, cultural, and academic aspects. In Jordan, some schools contract with private agencies for ABA services; however, neither consultee had experience working with ABA service providers. One teacher participant served as a knowledge expert. She was a Jordanian woman special education teacher with instructional expertise in Arabic and 16 years of experience (10 years as primary special needs coordinator at her current school and 6 years as an Arabic special education teacher). The other teacher participant served as the primary consultee. This teacher consultee was a female Jordanian Native-Arabic speaker teaching 6th-grade general education. She had 5 years of experience teaching Arabic, including 2 years at the current school and 3 years at a different IB school. Guided by cultural humility, the consultation process emerged through mutual engagement between the consultant and the Jordanian teacher. As part of this collaborative and cooperative process, the consultant reached out to the teacher consultee and invited her to identify a student in her classroom presenting behavioral concerns so the consultant could gain experience conducting the FACTS interview in Arabic. The consultant shared that she was enhancing her own consultation skills across language and cultural contexts but could also use this experience to provide ideas to the teacher consultee about how to understand student behavior functionally. The teacher demonstrated openness and buy-in by selecting a student whose behavior she was interested in addressing and talking through the consultation process (Goforth et al., 2025).

### FACTS interview form

**Figure 1:**
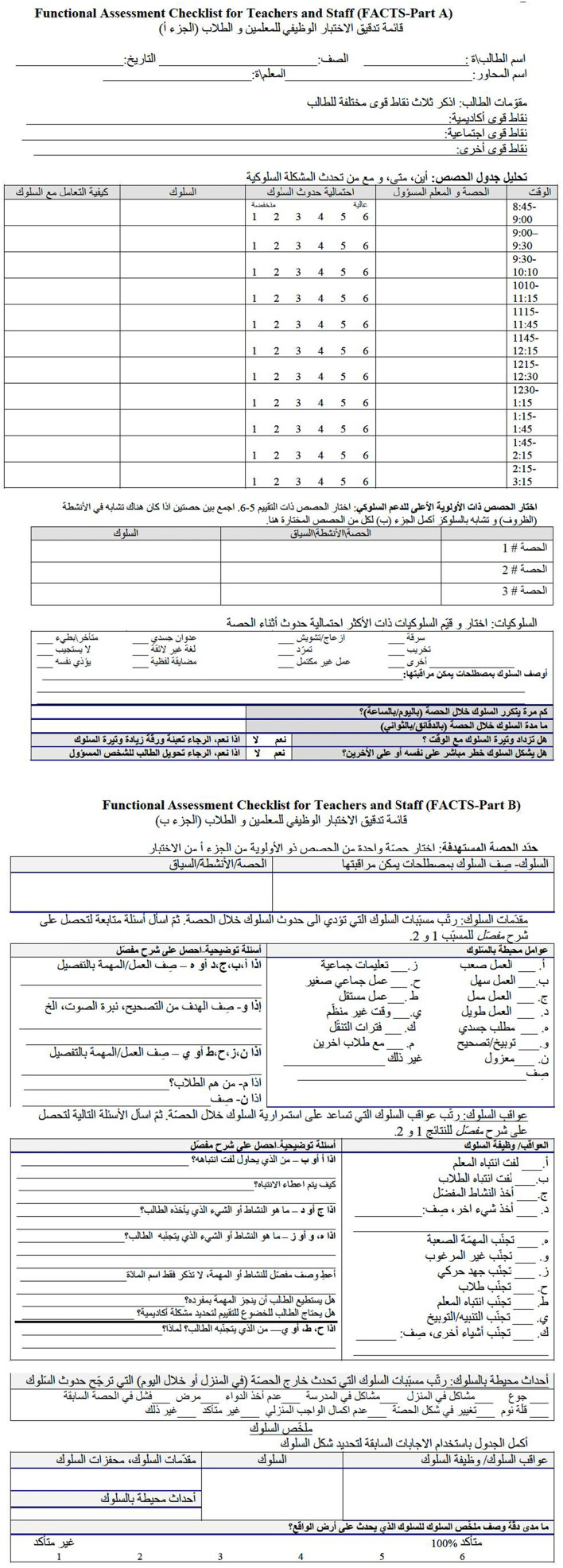
Arabic translation of the FACTS interview.

The FACTS is a semi-structured interview form that is designed to assess teacher perception of student behaviors and the environmental factors that contribute to occurrence and maintenance of behaviors (i.e., antecedents and consequences) to develop a functional hypothesis statement that can be used to inform additional data collection and ultimately behavior supports (McIntosh et al., 2008a,b). The FACTS interview form is freely available and regularly used in school-based FBAs. Despite the initial technical adequacy research and overall widespread use in practice, limited follow-up research has examined the FACTS interview systematically. Unpublished Spanish and Chinese language translations of the FACTS are available. However, there are no public (peer reviewed or unpublished) versions of the FACTS adapted for use in Arabic, and there is no information about the rigor or process for the unpublished Spanish and Chinese translations. In this research, the FACTS was translated into Arabic following ITC 2nd edition guidelines (2017), cultural humility, and Bernal's ecological validity model (Bernal et al., 2009). The adapted FACTS, presented in Figure 1, was then used to facilitate problem identification and analysis for FBA hypothesis development with a teacher working in Jordan.

### Supervision, reflective practice, and team-based analysis

The first author closely supervised the consultant during the teacher consultation case. Clinical case supervision followed traditional clinical supervision models of “do and review” with the addition of systematic prompting and consideration of cultural humility and adaptation models. The supervisor taught the consultant in previous coursework in behavior assessment and consultation, establishing the foundation for a strong supervisory relationship and the supervisor's understanding of the consultant's skills and knowledge related to behavior assessment and culturally responsive practice. Upon beginning the case study, the consultant met with the supervisor initially for 2 h to develop the scope of the work and discuss the process for obtaining research approval, consent, and steps for collaboration with two of the consultant's former teacher colleagues in Jordan. After this initial meeting, the consultant engaged in a four-quarter credit hour consultation-focused graduate class with the instructor weekly and met individually with the supervisor at least every other week outside of class time to review the consultation case progress, receive feedback, and discuss next steps. Some aspects of the consultation process were initially completed as assignments for the graduate class (e.g., reviewing or revising a consultation tool such as the FACTS and conducting a consultation interview) while other aspects (e.g., translation and adaptation) were supervised as part of the descriptive consultation case study only and not part of course requirements. Because all phases of the descriptive case were completed while the consultant was living in the US, consultation work was conducted remotely (via videoconferencing software).

Together and independently, researchers engaged in systematic reflective practice, data triangulation, peer and expert review processes, and team-based data analysis. In critical reflective practice, each researcher reflected before and after consultation interactions and data collection making certain to consider the accuracy, validity, and meaning of interactions and data. The supervising researcher and the consultant engaged in this process independently when reviewing/updating the FACTS form and via reflective journaling. The supervisor and consultant engaged in this process together during supervision when discussing the case. The third researcher provided data triangulation support and consensus by reviewing all data at the conclusion of the case and discussing the process and outcomes with the other researchers to ensure clarity and identify possible points of bias or misunderstanding. Each of these consideration points were then shared with research team members for member checking, consistency, and discrepancy. Some adjustments were planned based on the researchers' knowledge of school-based consultation and the Jordanian context while other adjustments were made in real time or in response to a discrepancy as it became obvious to one of the researchers.

Sometimes discrepancies emerged during the translation process but before the initial interview with the teacher consultee (e.g., such as how to operationalize culturally nuanced behavior terms or differentiate antecedents from setting events in Arabic or the need to adapt the routines section to align with Jordanian school schedules). In these cases, the supervisor and consultant reviewed the original English constructs, compared them to Arabic translations, considered cultural context, and collaboratively determined the most conceptually equivalent and culturally appropriate wording to include on the FACTS interview tool. During supervision, the consultant explicitly described these discrepancies verbally, using contextual and culturally grounded examples from Jordanian school settings to clarify meaning and illustrate how certain terms functioned differently across linguistic and cultural contexts. For example, she explained how particular behavioral descriptors carried moral or relational connotations in Arabic that differed from their technical behavioral definitions in English. This process allowed the supervisor to more fully understand the cultural nuances influencing translation decisions and provide feedback during individual supervision meetings. Revisions were documented and re-evaluated in subsequent supervision sessions. In other instances, the consultant engaged in a culturally responsive practice during a meeting (e.g., asking for open-ended responses rather than continuing to present a list when she observed teacher consultee behavior or responses that indicated dissonance).

The data sources involved in data triangulation for this case included all teacher consultation case data including the drafts of the translated FACTS interview tool in Arabic, completed FACTS interview notes, all primary teacher consultation meeting notes, language expert teacher consultee meeting notes, and consultant-clinical supervisor meeting notes. Team-based data analysis was conducted collaboratively between the consultant and the supervising faculty researcher. Following each major phase (i.e., initial translation, iterative adaptation meeting with the knowledge expert, and completion of the FACTS interview), the consultant engaged in structured debriefing meetings with the supervisor. During these meetings, the consultant presented translation decisions, cultural adaptations, interview excerpts, and emerging functional hypotheses. The supervisor systematically prompted reflection related to technical adequacy of FBA procedures, alignment with ITC translation guidelines (2017), and integration of cultural humility attributes (Foronda et al., 2016) and ecological validity dimensions (Bernal et al., 2009).

### Translation process and application of cultural frameworks for adaptation

The cultural humility framework (Foronda et al., 2016) was foundational to the translation and adaption process. Namely, the attributes of openness, self-awareness, egoless, supportive interactions, and self-reflection/critique were infused throughout consultation with the teacher and in clinical supervision. Throughout the consultation case, each aspect of the cultural and language translation process incorporated the cultural humility framework through both pre-planned and real time responsive integration (see Table 1). Consistent with practitioner-reflexivity (Brookfield, 1995), the consultant was trained to consider this framework and then the supervisor systematically prompted the consultant to identify relevant aspects at each phase in the process. Examples of this are integrated throughout the translation process, and Table 1 includes each attribute with Foronda et al.'s (2016) corresponding definition as well as examples of how each attribute was integrated in the current consultation case and a description of when/how each adaptation example was incorporated.

**Table 1:** Cultural adaptation examples of cultural humility attributes (Foronda et al., 2016).

Attribute	Definition	Examples of cultural adaptations	When and how adaptation considerations
Openness	Possessing an attitude that is willing to explore new ideas	1. Consultant initially watched others perform FACTS interviews to learn the skills. Consultant role played and obtained feedback 2. Consultant engaged in case supervision with research team member for critical reflexivity and cultural considerations 3. Translating the FACTS form to Arabic involved more than literal translation but also orienting text/visuals (right to left), adjusting the concept of routines analysis (every day is different in Jordanian school routines), operationalizing terms for target behaviors, and/or replacing them with familiar Arabic terms (e.g., disruption/annoyance)	1. Adapted in advance as part of initial learning 2. In advance, the supervisor and consultant discussed language and cultural adaptations, reviewed relevant cultural and language translation materials, and planned the project using these guidelines and frameworks. Throughout the supervision process, the supervisor prompted specific reflection for openness, and this occurred in real time during supervision meetings where supervisee questions were purposefully linked back to the frameworks 3. In advance, the consultant assessed for areas of language and contextual translation. In supervision, supervisor prompted for reflection and revision. Changes were incorporated in advance and made in real time during consultation meetings and interviews
Self-awareness	Mindful of strengths, limits, values, behavior, beliefs, and appearance	1. Consultant worked to improve communication by avoiding unconventional literal translations and jargon 2. Even though the consultant spoke Arabic, an Arabic-language teacher provided proofreading and suggestions for the translated version	1. In advance, this was discussed in supervision. During interviews in real time, consultant was attentive to consultee's body language and responses and adjusted language when disconnects seemed to occur. Supervisor pre-corrected for this and encouraged reflection during the process 2. In advance, the supervisor shared ITC guidelines, and the consultant was aware of her own language limitations and suggested a colleague who could be an expert
Egoless	Being humble, viewing the worth of all individuals on a horizontal plane	1. Consultant asked the teacher to describe behavior in her own terms rather than picking from a list 2. Consultant was aware that behaviors, values, and beliefs that fall outside the mainstream can be interpreted as within-child problems when, instead, they are differences rather than deficits. The teacher stated that the student is behaving this way because “he is an only male child, and his parents spoils him.” Consultant listened without judgment of the teacher while keeping this perspective in mind for future interactions	1. In advance, the possible challenge with the list of items was pre-identified in planning, and some modifications were made to the list prior to the interview. However, switching to open-ended questions was not preplanned but was a real time response to the teacher's hesitation about picking from a list and her questions about what certain terms on the list meant, consistent with Jessel et al. (2025) recommendations 2. This was taught in literature broadly on implicit bias and the need for operationally defining concerns. Pre-planned to acknowledge and respect cultural values and beliefs of consultee (Almarzooq et al., 2025; Fisher, 2020). This specific response was not pre-planned and occurred in real time, illustrating the consultant's knowledge of a culturally responsive practice (e.g., engage in egoless) during interviewing
Supportive interactions	Intersections of existence among individuals that result in positive human exchanges	1. Did not video record the interview because it was conducted after school hours, and the teacher was not wearing her hijab at that time 2. Consultant interviewed teacher in Jordan using Arabic. Consultant used colloquial Arabic (specific to the Jordanian dialect) to explain the meaning of words that were difficult to understand 3. Operationally defined behaviors in the Jordanian context to better explain or ensure understanding of	1. This response occurred in real time. Although a recorded interview would have allowed for a stronger study, the consultant recognized the cultural values and comfort of the consultee in the moment and prioritized this over her own goals 2. This was both pre-planned during supervision time and responsive in real time since the consultant anticipated some (but not all) of the terms that might need additional explanation
		the concept in the context (ecological translation beyond language translation and provided open-ended prompts rather than lists to better understand the teacher's values and classroom expectations from a cultural lens	3. Like the second example, in that some aspects were planned based on knowledge of FBA interviewing, Arabic language, and Jordanian culture. However, the pivot to open-ended prompts was made in real-time interviewing
Self-reflection/critique	Critical review of one's thoughts, feelings, and actions	1. Consultant engaged in reflective journaling to better understand their own cultural lens 2. Consultant asked for feedback from mentors when learning and revising the FACTS interview and from consulting teachers	Both items were expected and planned during supervision to support critical reflection and to promote the consultant's social-emotional learning skills in reflection, which is a training standard for the consultation course

#### Initial translation

The first step in using the FACTS interview in Jordan was to translate the tool from English to Arabic using ITC 2nd edition (2017) best practice guidelines for initial, forward, and backward translation all within the context of cultural humility and Bernal et al.'s (2009) ecological validity process for making cultural adaptations. Because the FACTS is a free use interview, permissions were not needed prior to translation. The consultant who is fluent in Arabic language and culture as well as her supervisor reviewed research in consultation and behavior analysis in Arab regions to establish pre-consideration construct equivalency and overlap. The consultant also collaborated with the teacher participant who served as the knowledge expert in Arabic language and culture who also had professional expertise in education as an English teacher in Jordan. These activities were pre-planned at the onset of the case according to the translation guidelines and cultural humility framework.

The consultant (a native Arabic speaker) used Modern Standard Arabic (MSA) to conduct the initial forward translation from English to Arabic. MSA is the formal version of Arabic used in writing, news, and literature across the Arab region. The initial linguistic translation process was literal to maintain the originality and objectivity of the English version of the FACTS, but this was challenging. One difficulty was the orientation of the interview form. Since Arabic is read from right to left, the layout of the document had to be adjusted to maintain readability. This required reordering tables and text boxes so they would be intuitive for Arabic-speaking users while keeping with the original structure of the FACTS (i.e., the routines analysis section was especially challenging to visually orient). Additionally, one section of the form instructs teachers to complete a behavior escalation worksheet if a student's behavior intensifies. This section was culturally irrelevant in the Jordanian school context, as behavior escalation procedures and documentation methods differ dramatically. To ensure the translation remained functional for its intended audience, this section was initially left as a literal translation to be discussed with the Jordanian teacher for input on adaptation to the Jordanian school context. As the consultant initially translated the FACTS, another unanticipated significant challenge arose with specific terminology requiring real-time openness and self-reflection. For example, the terms “antecedents” and “setting events” translated into the same Arabic word, and the consultant recognized the limitations of translating these to the same word from a technical aspect of behavior assessment interviewing. In English, setting events refer to events that increase the likelihood of a behavior occurring (e.g., lack of sleep or changes in routine), while antecedents are immediate triggers (e.g., being given a difficult task). Without distinct Arabic equivalents, additional cultural clarifications (e.g., Arabic example prompts to distinguish the terms) were necessary to differentiate the two concepts within the translated document. The consultant embraced openness from the cultural humility framework during this phase as she initially conducted literal translations and considered her own awareness (including training in behavior assessment in the US and using English terms) and challenges of extension to Jordanian school contexts.

#### Translation iteration

After the initial forward translation, the Arabic-language teacher participant served as the knowledge expert reviewer and a secondary linguistic and cultural translator, illustrating the consultant's engagement in being egoless. The consultant sent both the English FACTS form and the MSA translated version to the teacher participant serving as the knowledge expert by email a week before a scheduled video meeting to discuss the FACTS. The knowledge expert reviewed the documents prior to the meeting. During the meeting, they placed the forms side-by-side for comparison and engaged in a backward translation process where questions were translated back to English. The resulting changes were specific to the terms used to describe the target (i.e., “problem” behaviors), which were significantly revised due to the unclear literal translation of behaviors that were not present in Jordan or the Arabic language context. For instance, the word “disruption” (خلل) (Khalal) is widely used in the American school context. However, when used in the Jordanian context, it was replaced by the literal translation of the word “annoyance” (إزعاج) ('izeaj) to match the language spoken by most teachers who would not have knowledge of the literal translation for “disruption.” Other example behavior terms were replaced by synonyms to match the Jordanian school context. At the conclusion of the meeting, the knowledge expert and the consultant finalized the translated version for practice use with the other teacher consultee participant who worked in the same school in Jordan.

Upon completion of the cultural and linguistic translation iterations, the consultant reflected on the cultural humility framework in relation to the translated FACTS form with reflective journaling and verbally with the supervisor as the consultant prepared to interview the primary teacher consultee. The consultant engaged in self-reflection and debriefed with the clinical supervisor regarding the application of the cultural humility framework to the process. The consultant and supervisor reviewed the new changes resulting from the first two translation phases, discussing the impact on the FBA interview, and used this information to support the consultant in preparing for the interview. This iterative process of linguistic forward and backward translation and cultural adaptations, combined with critical reflexivity after each interaction, persisted throughout the consultation case study before the culturally and linguistically translated interview was field tested (i.e., conducted) with the teacher consultee.

### Conducting the translated FACTS interview and iterative adaptation

After the initial translation was complete, the consultant reached back out to the primary teacher consultee to schedule an interview. The consultant reminded the teacher that the discussion was a practice opportunity to expand the consultant's knowledge and skills in behavior assessment and consultation across cultural and language contexts and could result in providing some ideas for how to conceptualize or support a student's behavior in the teacher consultee's classroom. The consultant asked the teacher to focus on a student who exhibits “problem” behavior in her classroom. The teacher consultee reconfirmed her willingness to participate and agreed to meet via video conferencing to discuss her perception of a student's behavior in detail. The consultant was familiar with the teacher's school setting but did not know the target student for the FACTS interview or have any contact with the current classroom students or setting since she was in the US during this time.

The consultant approached the interview with openness and flexibility while she conducted an hour-long initial interview with the teacher colleague. She engaged in a pre-planned supportive interaction by scheduling the interview outside of the school day at the teacher consultee's request. An additional real-time supportive interaction with collaborative posturing and a consultee-centered approach occurred when the consultant initially planned to record the video conference interview for reflection but did not record. This change occurred because when she asked the teacher consultee about recording the interview, the teacher expressed discomfort with recording since she was not wearing a hijab, and she would feel uncomfortable video recording without a hijab.

### Interview process outcomes

#### Setting the stage during the interview

At the beginning of the interview, as a pre-planned supportive interaction, collaborative posturing, and consultee-centered approach, the consultant asked the teacher about her familiarity with the consultation process. The teacher responded that she had some experience working with school specialists to support students with behavioral challenges but had never participated in a formal FBA or an individualized FBA interview. The consultant asked about the teacher's familiarity with behavioral problem-solving strategies, to which the teacher explained that she primarily relied on classroom management techniques such as redirection and reinforcement but was not accustomed to systematically analyzing behaviors based on their functions.

Next, as pre-planned supportive interactions, the consultant provided an overview of how their consultation would unfold in relation to the typical purpose of the FBA process and a FACTS interview. The consultant reminded the teacher consultee that she should ask questions and seek clarification throughout, and she was also reminded that she would contribute to the knowledge-making process as they collaborated and jointly examined terms and concepts for adaptation. The consultant then explained that the purpose of the FBA was to identify patterns in the student's behavior, determine what might be triggering and reinforcing it, and use this information to help develop effective interventions. The consultant also emphasized that the goal of FBA was not just to describe what the student was doing but to understand why the behavior was occurring so that the teacher consultee could shift from seeing the student's actions as personality-driven to viewing them through a functional lens. To achieve this, the consultant provided an overview of the functions of behavior, explaining that students engage in behaviors to obtain something (e.g., attention or something tangible) or to avoid something (e.g., difficult tasks), followed by explicit and systematic (e.g., after each component description) checking for understanding, cultural relevance, and equivalence.

#### Conducting the interview

At the outset and throughout the interview, the consultant attempted to remain egoless and encouraged the teacher to ask for clarification at any moment and ask for additional examples and terms as needed, ensuring she fully understood each section of the FACTS. The consultant observed the teacher as carefully as possible to identify areas of possible confusion or bias and approached those interactions supportively, self-reflecting on her own experiences of first engaging in FBA interviewing a few months prior as a graduate student. Given the teacher's cultural context of a Jordanian school setting, the consultant was mindful of norms that typically shape such conversations in educational settings, including indirectness when discussing sensitive topics. For example, teachers may soften or “sugar-coat” descriptions of challenging behavior to avoid perceived criticism of the student or family. As a result, the consultant pre-planned to frequently invite elaboration through follow-up questions and requests for concrete examples to ensure a complete and accurate understanding of the student's behavior. Although follow-up questions to operationalize behaviors are important in Western contexts as well, this was even more critical in the Jordanian context. It was important that the consultant navigate this follow-up with real-time responses, showing care and supportive interaction, since asking for clarification that leads to direct behavior descriptions was likely an unfamiliar (and possibly uncomfortable) way of discussing student behavior for the teacher in the case study.

The consultant planned to attend to the collectivist orientation of the culture, in which student behavior is often viewed within the broader family and social system rather than as a discrepancy between the individual student's skills or behaviors and the expectations of their educational environment. Although the consultant was unclear on how this might surface when planning the interview, she quickly observed when the teacher described the student's behavior in relation to family practices and dynamics, at times positioning the family as contributing to the student's challenges (e.g., suggesting the student is “spoiled” and therefore misbehaving). Based on her pre-planned approach, the consultant deliberately chose to remain egoless and approached the discussion with supportive interaction rather than dismissing the teacher's viewpoint. The consultant acknowledged the responses respectfully and noted the relevance within a collectivist framework while also gently guiding the discussion back to observable school-based behaviors and environmental factors (e.g., antecedents/consequences) to align with FBA best practices.

The consultant was self-aware of the predominance of Western perspectives and understandings regarding the questions on the FACTS interview as well as the teacher's language dialect, so she pre-planned the use of colloquial Arabic (specific to the Jordanian dialect) to explain the meaning of words to support the teacher's understanding and increase her comfort in the interview. Even then, the consultant had to remain in a supportive role and continuously self-reflect in real-time during the interview to balance rapport and cultural comfort with the technical aspects of FBA interviews. As another example, the content of the FACTS interview followed the typical organizational interview flow and started with a discussion of student strengths. However, the teacher seemed unprepared to discuss strengths and initially had some difficulty in pinpointing strengths. The consultant was able to quickly recognize this in real time and prompted for academic strengths specifically and then circled back to social skill strengths, which were more difficult for the teacher to identify. The teacher was then able to respond with strengths, sharing that the student had strong reading comprehension skills, had several friends (despite his behavioral challenges), and was always willing to take on a leadership role or classroom job.

### Identifying and operationalizing behaviors and environmental variables

When asked about the school day (routines), the teacher expressed difficulty with the routines section as it is in the original FACTS form. This is because the school day in the US typically follows a consistent schedule, with students attending the same classes at the same times each day. However, in Jordan, the school schedule varies daily, with subjects and activities given at different times. This structural difference required adjustments to the FACTS interview form to ensure relevance and applicability to the Jordanian school system. Although the consultant was knowledgeable about this deviation from US norms, her experience with the interview tool in the US led to an initial oversight about this, and she did not pre-plan the framing of this part of the interview for the Jordanian context. However, because the consultant engaged in regular reflection during the interview, she quickly recognized the need to accommodate this in a supportive way and, in real-time, adapted the routines section of the FACTS interview to discuss student behavior in relation to specific activities rather than a schedule following fixed time periods. This allowed for an analysis of student behaviors across routines but not necessarily at fixed times in the day.

Next, the consultant allowed the teacher to read the list of target behaviors and ask for clarification for any that were unclear, and it was apparent that there were multiple terms that were not clear. The consultant initially responded by operationally defining target behaviors in Arabic for additional clarity to increase the relevance of each concept in the context (i.e., ecological translation beyond language translation). The consultant, recognizing the need for a culturally responsive egoless approach, made a deliberate shift from the translated FACTS interview structure in real time. She asked the teacher to describe the student's behavior in her own words instead of choosing from the list and then matched the teacher's description with the pre-listed behaviors that best fit the operational definitions. Because this approach worked well for defining the behavior, the consultant continued with the process of asking the teacher to identify rather than select for determining possible antecedents, consequences, and setting events. This appeared to work well, and the interview resulted in a logical hypothesis statement. This nuanced but important real-time change from providing a menu of choices to more open-ended responses with follow-up questions to clarify seemed to be an important critical reflective practice that allowed for increased cultural relevance while maintaining technical adequacy of the interview.

The collectivist context and the resulting tendency of the teacher consultee to attribute student behavior to poor upbringing rather than situational or instructional factors were another challenge in the adaptation process that impacted both the consultant's ability to obtain operational definitions and permeated throughout the interview. Like the impact in the operational definition conceptualization, the notion in many Arabic-speaking cultures that behavior is understood through a moral lens, where misbehavior might be seen as indicative of family values rather than as a response to environmental contingencies, persisted throughout the interview. To overcome this, the consultant pre-planned strategies grounded in the “Consultation Through a Cultural Lens” framework, which emphasizes reframing issues to align with culturally relevant constructs. The consultant provided intentional and responsive supportive interactions validating the teacher's concerns while gradually and delicately redirecting the discussion toward observable behaviors and environmental events. Near the conclusion of the interview, the consultant formulated a hypothesis statement in Arabic that incorporated all relevant factors the teacher shared influencing the student's behavior, and the teacher confirmed that the behavior summary was accurate. The teacher asked about it and the consultant provided some functional behavior support strategies aligned to the hypothesis of the student's behavior and scheduled a follow-up in 2 weeks to assess the teacher's perception of the process and outcomes.

#### Follow-up post interview

After 2 weeks, the consultant emailed with the teacher consultee who reported using the strategies and an increase in the student's expected behaviors. The teacher also shared she would appreciate more access to similar assessments and positive behavior supports through her school system, further supporting this culturally centered consultation process. Notably, the teacher demonstrated acceptance of a behavioral hypothesis that differed from common societal and cultural approaches to problem identification in Jordanian contexts, where student behavior is often attributed to family practices or individual deficits. Instead, the teacher embraced a more detailed, contextualized, and function-based understanding of behavior that emphasized environmental variables and instructional support within the classroom. It is hypothesized that the collaborative and culturally responsive and reflexive approach, combined with the educational context in Jordan for supporting student behavior needs, played a significant role in tailoring the strategies to fit the cultural and contextual needs of the classroom.

## Consultation case summary and conclusions

The case study showed that the culturally and linguistically adapted FACTS interview was accessible and relevant to a Jordanian teacher, though it required both significant language translation and additional follow-up questions and examples connected to local classroom routines. The interview adaptation showed cultural differences in how teachers operationalized behaviors, considered routines, understood consequences and motivations for student behavior, and how factors like social respect, peer dynamics, and family expectations are viewed within the Jordanian educational context. The results also showed that while the translated tool held initial promise for validity and acceptability, its effectiveness depended on pairing the translated FACTS interview form with a consultant engaging in and committed to culturally reflective consultation and training in behavior assessment. Therefore, the success and effectiveness seen with this consultation case may depend largely on the technical training and background of the consultant (e.g., training in behavior assessment), their knowledge of the cultural context where they are working, and their knowledge of cultural adaptation and responsiveness models. Access to supervision to support pre-planned and real-time culturally responsive and reflective responses may also impact the success of this work. This combination illustrates a process that can be replicated to further evaluate language and cultural translation of school-based behavior assessment tools such as the FACTS interview. Culturally and linguistically adapted tools can then be systematically analyzed and used to inform technically adequate and culturally acceptable hypothesis development to guide behavior intervention planning.

## Discussion

Throughout the process, the researchers aligned their work to established guidelines and frameworks for translation and adaptation of assessment tools. ITC 2nd edition guidelines were followed as much as possible, and a multicultural consultation lens (i.e., Ingraham, 2000; Sheridan, 2000) and the cultural humility framework (i.e., Foronda et al., 2016) were applied to understanding the different perspectives of stakeholders. The supervisor and consultant purposefully planned an iterative process utilizing the ITC guidelines and the frameworks to support linguistic and cultural adaptations. The consultant had graduate coursework in the US using Western-derived behavioral assessment and consultation frameworks prior to beginning the case, and she then worked carefully with her supervisor to conceptualize the process for cultural and linguistic translation. Throughout the process, she engaged in independent reflection via journaling and utilized ongoing supervision to support her skill development and reflection. These strategies likely reinforced real-time cultural responsiveness during the interview. Lastly, all three researchers engaged in the post-process reflection and data triangulation after the interview's completion to further analyze the process and outcomes.

The language and cultural match between the consultant, teacher, and student likely influenced this process, but the exact influence is unknown. It is possible that the shared cultural background resulted in assumptions and understandings that were not illuminated in this work due to the collaborative understanding of variables like the Jordanian school context and the Arabic language. It is also possible that the shared cultural match was less strong in this case due to the consultant's training in Western-derived behavioral frameworks that are different from typical frameworks for understanding behavior in Jordan. Sometimes cultural and contextual diversity can exaggerate or highlight areas of disconnect that should be explored. Conversely, the shared background may enhance the consultant's understanding of cultural influences, and this may improve the consultant's ability to anticipate and prevent or efficiently respond to initial barriers with language and cultural translation. In this case study, the teacher consultee and consultant shared a common language and cultural background and had a prior collegial relationship, which seemed to support rapport building (Sheridan, 2000). The shared racial ethnic background between consultant and consultee may ease the development of a collaborative and participatory process, as the shared background could increase trust in the consultant, allowing the consultee to more easily view the consultant as a meaningful contributor to the process (Goforth et al., 2025). For example, in this case, sharing a cultural background possibly increased the likelihood that the consultant was able to quickly understand and acknowledge the teacher's belief that the student's behavior was influenced by his status as the only male child in the family. This shared cultural background likely decreased the chances of missing or dismissing the comment and instead allowed for an opportunity for the consultant to validate the teacher's perspective while gently steering the discussion toward the consultant's behavioral framework to understand environmental and functional assessment of the behavior. Although language and ethnic cultural match may not always be possible or may not always positively influence the outcome, contextual and cultural match has been shown to enhance consultation outcomes (Goforth et al., 2025) and should be considered a possible influential variable.

The incorporation of the cultural humility framework from the onset likely allowed for contextual translation that transcended limitations associated with literal translation and may reduce some consultant biases. This framework was enacted as a “way of being” and illustrated practitioner reflexivity by engaging in lucid adaptation prior to, during, and following the interview. The clinical supervision that supported continuous critical practitioner reflexivity and systematic considerations for the researcher-practitioner privilege, position, and power (Brookfield, 1995) was likely heavily influential in the process, as was the supervised support regarding the technical aspects of functional behavior assessment. However, the exact impact of supervision in the process is unknown, and there may be some aspects of this work that apply across contexts, no matter the cultural context. For example, operationalizing target behaviors and environmental variables is a challenge in any context, as the interviewing consultant must understand what a teacher means when using descriptive terms for behaviors, and many teachers, not just those in a Jordanian context, may bring biases about the function of behavior that are different from the environmental variables explored in a behavior analytic approach.

Lastly, although there were moments where the teacher seemed somewhat resistant to Western ideas of behavior, the consultant was able to guide her to a functional behavior hypothesis that the teacher rated as acceptable and accurate. Even though direct data were not collected on the teacher or the student, the process seemed feasible and appropriate as indicated by the teacher's agreement to engage, openness to the discussion, and acceptance of the final hypothesis statement as valid. She also asked for input to support the student's behavior, stated she implemented corresponding strategies even when not required as part of her professional work, and followed up again to indicate she wished more similar supports were regularly available through her school directly.

### Limitations

Several factors limit the implications that can be drawn from the current work. First, although pre-testing or field testing of linguistically and culturally adapted methods is a critical step in translating materials, this was an initial exploratory descriptive translation and adaptation case study with only one teacher/consultant dyad. Systematic behavioral data collection, psychometric validation, and structured outcome evaluation were not conducted. Therefore, this case only illustrates one example of a process and does not yet provide evidence for the approach or outcomes. Next, the consultant had a unique background as a clinically supervised graduate student researcher-practitioner with specific expertise in applied behavior analysis, lived experience in Arabic language/culture, prior history as a special education teacher in Jordan, prior history working with the teacher consultee in the study, strong knowledge of the English language, and graduate education in the United States. Consultants with different backgrounds or without clinical supervision may have different experiences creating or using a translated FACTS interview form, or the translation process and outcomes may not convert similarly to other international contexts. Additionally, the translation process is limited by the unique nature of this case in the Jordanian school context. The progress in Jordan regarding the use of behavior analysis and the specific school context where the participants worked may have influenced the process (i.e., more readiness to engage in functional behavior assessment and ways of thinking/discussing classroom behaviors). The teacher and the consultant were previously colleagues, so initial trust and the teacher's willingness to “help” the consultant in her learning may have positively skewed or otherwise influenced the outcomes. Another limitation is that although the study relied on an Arabic language teacher for expert review, all participants primarily spoke the same dialect of Arabic, which may limit usability if participants speak in different dialects. Using a teacher as the language expert may also influence the results since the teacher's knowledge of the school context and the terminology related to school-based assessment and consultation could influence the translation of educational jargon. Additionally, all were fluent in written Arabic. Newer Arabic speakers may benefit from additional scaffolding in the written language to enhance understanding. Similarly, the consultant recognized she sometimes used her English knowledge to differentiate similar Arabic words (e.g., antecedent vs. setting event), and it is unknown how someone who did not understand the terms in English would overcome this challenge. Lastly, direct data were not collected on the teacher or the student, so the actual impact of using the assessment to develop a hypothesis statement and corresponding support plan is unknown.

### Implications and future research

The findings from this researcher-practitioner-led exploratory descriptive teacher consultation case study provide new knowledge that enhances and extends functional behavior assessment interviewing to a broader international context. Future studies can replicate the integration of the International Test Commission guidelines, Ingraham's multicultural consultation framework, and Foronda's cultural humility framework into assessment and consultation translation. Future studies can examine different approaches to translation that incorporate these frameworks and can engage in systematic analysis to validate the findings with different consultation tools and broader participant samples. With regards to the FACTS translation specifically, future studies could integrate more participants from a broader range of backgrounds and different school contexts to better understand translation implications and systematically analyze the psychometric findings. Additionally, future work can integrate direct assessment of teacher and student perceptions and behaviors to increase objectivity in consultee and student outcomes because of this work. For future work in Arabic language translation contexts, it will be helpful to include participants who do not also speak English (e.g., to better understand how to distinguish/cue similar terms without referencing them in English), who speak different dialects, or who are still developing Arabic language skills to understand how scaffolding (e.g., using Arabic accents to support reading) and dialect/translation adjustments may impact the process. Broader implications for assessment and consultation are also important. This work illustrates the role of cultural adaptations beyond literal language/linguistic translation and the possible value of incorporating clinical supervision that employs a clear, critically reflective practitioner orientation, such as that provided by the cultural humility framework, as well as a clear framework for behavior assessment. It may be helpful to examine the role of supervision (and group vs. individual reflection in the process) in the linguistic and cultural translation process. Additional research in this area will likely illuminate important nuances in cross-cultural consultation that are unable to be surfaced in a descriptive case study. The current study is one that may pave the way for additional work that incorporates the cultural humility framework as well as highlights learning through diverse cross-cultural and language lenses represented in the researchers' identities to enhance educational and behavioral health outcomes.

## Data Availability

The original contributions presented in the study are included in the article/supplementary material, further inquiries can be directed to the corresponding author.
